# Time

**Published:** 1873-01

**Authors:** 


### The Bistoury.

ELMIRA, N. Y., JAN., 1873.

The BISTOURY is published Quarterly, upon the 1st of April, July, October and January, at Fifty Cents a year, in advance.

### TIME.

Another year of editorial labor has passed for us, and we are again found penciling our yearly salutation to our subscribers—reminding them that their subscriptions have expired, and that another payment is now due.

We hope all will not only find it convenient to return the enclosed envelope and blank, properly filled out, containing also the necessary fifty cents, but will consider it a pleasant duty so to do. We have afforded you a neat little magazine, brim-full of useful information, at an exceedingly small subscription price. We hope we have made it so interesting and desirable, that it will be difficult for you to give it up, and that you will always be found upon our list, duly checked, as a paid-up subscriber.

Many are yet owing us for four years subscription—ever since the BISTOURY has been issued in magazine form, and pay demanded for it. For all such, we enclose a suitable blank, and hope they will at once pay our claim upon them, and order the journal discontinued, if they do not desire to pay for it; otherwise, we will be compelled to force payment, when the sum has grown sufficiently large to warrant such a procedure.

Our next number will be the beginning of volume nine—the ninth year of the existence of the BISTOURY! We begin to feel almost like a veteran in the editorial chair, and hope that our experience will teach us how to increase the usefulness of our journal in the future, as it has been our effort so to do, in the past. Wishing all our patrons good health, continued prosperity, and a happy new-year, we submit the last number of volume eight for their inspection and approval.

